# Focused ion beam processing to fabricate ohmic contact electrodes on a bismuth nanowire for Hall measurements

**DOI:** 10.1186/1556-276X-8-400

**Published:** 2013-09-26

**Authors:** Masayuki Murata, Yasuhiro Hasegawa

**Affiliations:** 1 Saitama University, 255 Shimo-okubo, Sakura-ku, Saitama 338-8570, Japan; 2 Japan Society for the Promotion of Science, Tokyo, Japan

**Keywords:** Bismuth nanowire, Hall measurement, Focused ion beam, Ohmic contact, Thermoelectrics

## Abstract

Ohmic contact electrodes for four-wire resistance and Hall measurements were fabricated on an individual single-crystal bismuth nanowire encapsulated in a cylindrical quartz template. Focused ion beam processing was utilized to expose the side surfaces of the bismuth nanowire in the template, and carbon and tungsten electrodes were deposited on the bismuth nanowire *in situ* to achieve electrical contacts. The temperature dependence of the four-wire resistance was successfully measured for the bismuth nanowire, and a difference between the resistivities of the two-wire and four-wire methods was observed. It was concluded that the two-wire method was unsuitable for estimation of the resistivity due to the influence of contact resistance, even if the magnitude of the bismuth nanowire resistance was greater than the kilo-ohm order. Furthermore, Hall measurement of a 4-μm-diameter bismuth microwire was also performed as a trial, and the evaluated temperature dependence of the carrier mobility was in agreement with that for bulk bismuth, which indicates that the carrier mobility was successfully measured using this technique.

**PACS:**

81.07.Gf

## Background

Bismuth nanowires are widely known as suitable materials for quantization because bismuth has a very long Fermi wavelength and mean free path length of carriers and phonons [1,2]. Therefore, it is expected that one-dimensional density of states will be observed on a larger scale than other materials. Furthermore, it is predicted that the thermoelectric performance of bismuth nanowires as a one-dimensional geometry will be enhanced with a diameter of less than 50 nm due to semimetal-semiconductor (SM-SC) transition [3-5]. Many researchers have reported the thermoelectric properties of bismuth nanowires fabricated using various methods [6-14]. Our group has successfully fabricated a quartz template with a hole diameter of several hundred nanometers by applying the fabrication technique for optical fibers. Bismuth nanowires over 1 mm long and with diameters of several hundred nanometers have been fabricated by injecting molten bismuth into the nanohole at a high pressure of almost 100 MPa and then recrystallizing the bismuth by reducing the temperature [15]. The fabricated bismuth nanowires were identified as single crystal from X-ray diffraction measurements [16] and Shubnikov-de Haas oscillations [17]. To measure the resistivity and Seebeck coefficient of the nanowires, titanium (Ti) and copper (Cu) thin films were deposited on the edges of the bismuth nanowire to obtain appropriate thermal and electrical contacts [18]. The resistivity, Seebeck coefficient, and thermal conductivity of the bismuth nanowires and microwires (300-nm to 50-μm diameter) were successfully measured using this technique [15-25]. The temperature dependence of the Seebeck coefficient and electrical resistivity for bismuth nanowires with diameters smaller than 1 μm are completely different from those of bulk. Size effects in bismuth appear for larger size samples than other materials because the mean free path length of the carriers is very long and in the order of several millimeters at liquid helium temperatures. Furthermore, calculation models with three-dimensional density of states for the thermoelectric properties of bismuth nanowires have also been established [26-30]. The results have suggested that the carrier mobility is decreased with a reduction of the wire diameter due to the limitations placed on the mean free path by narrowing. This was confirmed using an evaluation model for measurement results of the resistivity and Seebeck coefficient [15,22]; however, direct measurement of the carrier mobility, such as Hall effect measurements, has not yet been performed. There have been very few reports on Hall measurements in the field of nanowire studies due to the difficulty of electrode fabrication on such a small area [31], and there have been no reports on such with respect to bismuth nanowires. There have been various reports on the temperature dependence of the electrical resistivity and Seebeck coefficient for bismuth nanowires, although it has been unclear why there are inconsistencies in these reports [6-12]. Our previous study revealed that the thermoelectric properties of bismuth nanowire are strongly dependent on the crystal orientation of bismuth, due to its anisotropic carrier mobility [23]. The next step is direct measurement of the carrier mobility by Hall measurement for bismuth nanowires with diameters of several hundred nanometers; however, it is challenging to fabricate electrodes on the surface of a bismuth nanowire that is encased in a template. We have previously reported the successful fabrication of electrodes on a bismuth nanowire encased in a quartz template by utilizing a combination of chemical mechanical polishing (CMP) and focused ion beam (FIB) processing. The resistivity of the bismuth nanowire was thereby successfully measured using the four-wire method [32]. As a next step, a technique for exposure of the bismuth nanowire for Hall measurements was also developed [33]. Many researchers have reported the resistivity of bismuth nanowires measured using the two-wire method due to difficulty of electrode fabrication with the four-wire method; however, the four-wire method is theoretically more suitable for estimation of the resistivity. There have been some results reported for the resistivity measured using the four-wire method; however, the surface of bismuth nanowires is oxidized during the fabrication process, which makes it difficult to fix the boundary conditions for the wire diameter direction [12-14]. Furthermore, it was reported that a majority of the bismuth nanowire becomes amorphous due to irradiation with a high-energy gallium (Ga) ion beam during FIB processing [13]. Therefore, it would be difficult to successfully apply FIB processing to a bare bismuth nanowire. However, the bismuth nanowires prepared in our work were completely encased in a quartz template. Therefore, the influence of Ga ion beam irradiation could be neglected if the exposed area was very small with respect to the entire surface of the bismuth nanowire. The FIB processing technique was applied to fabricate electrodes on a 521-nm-diameter bismuth nanowire for Hall measurements, and the electrodes were evaluated to confirm a suitable contact. Furthermore, the temperature dependence of the resistivity was measured with comparison of the two-wire and four-wire resistance measurements. To confirm the validity of the electrode fabrication technique to estimate the Hall coefficient, Hall measurements were performed using a 4-μm-diameter bismuth microwire. It would be ideal to use a nanometer-order diameter wire to demonstrate the Hall measurement; however, verification with a 4-μm-diameter microwire was performed first, which is predicted to give almost the same Hall coefficient as that of the bulk. We discuss the adequacy of the electrical contacts on the bismuth nanowires for resistivity and Hall measurements.

## Methods

**Figure 1:**
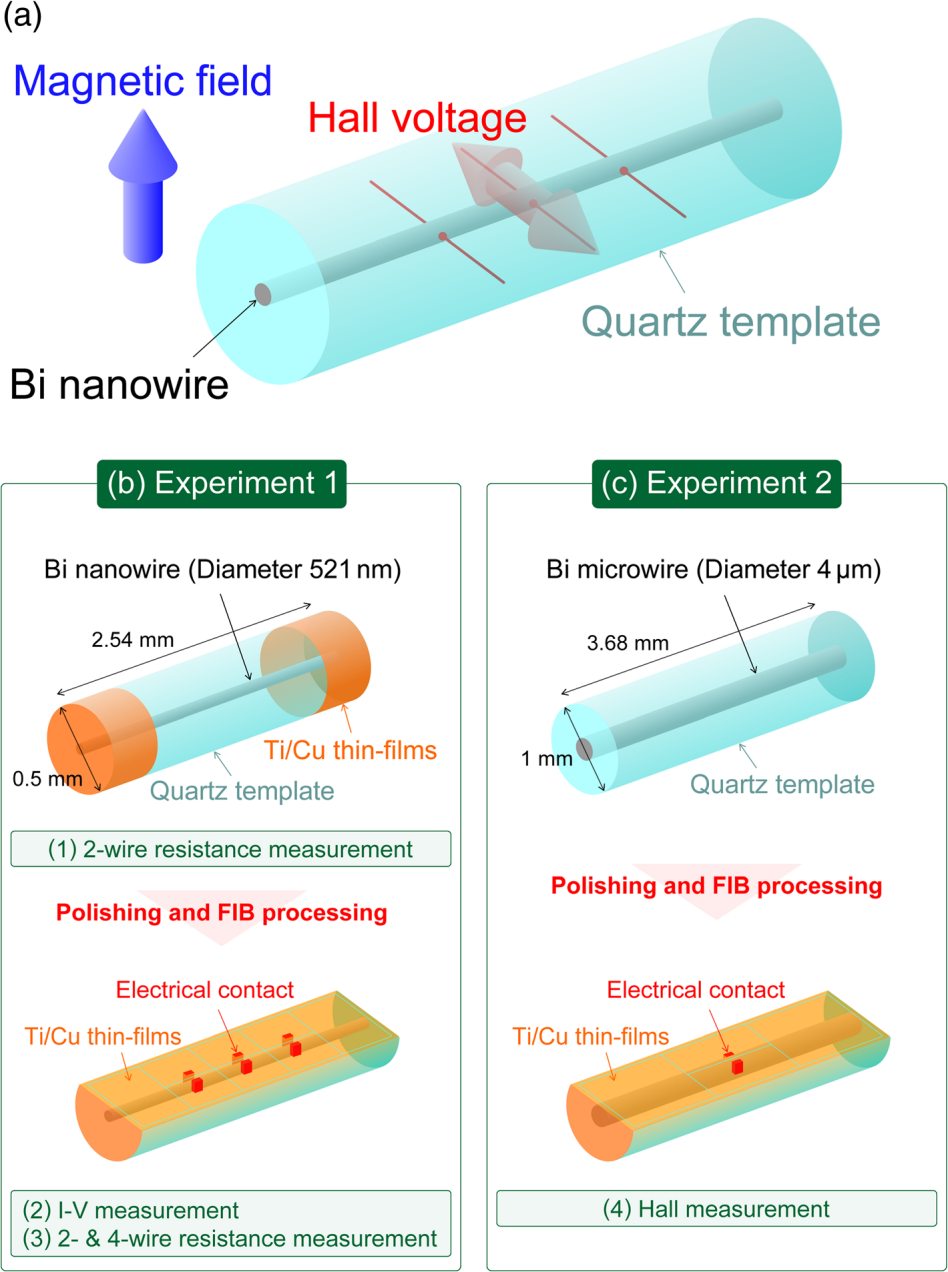
**Experimental procedure for the fabrication of electrodes and evaluation of bismuth nano- and microwires. (a)** Configuration for Hall measurements of a bismuth nanowire. **(b)** Procedure for the fabrication and evaluation of electrodes on a 521-nm-diameter bismuth nanowire. The two-wire resistance was measured before FIB processing, and the *I*-*V* relationship and two- and four-wire resistance were measured after FIB processing. **(c)** Procedure for the fabrication of electrodes for Hall measurements.

Figure 1a shows a schematic diagram of the configuration used for Hall measurements of bismuth nanowires. Although electrodes are required on the side surfaces of the bismuth nanowire for Hall measurements, these bismuth nanowires are covered with the quartz template, as shown in Figure 1a. Therefore, polishing and FIB processing were applied to fabricate the electrodes for Hall measurements. The experimental procedures are presented in Figure 1b,c. Two bismuth wire samples were employed: a 521-nm-diameter nanowire for evaluation of the electrical contact to establish a suitable technique for the fabrication of ohmic contact electrodes (experiment 1), and a 4-μm-diameter microwire for Hall measurement to determine whether Hall measurements could be successfully performed with this technique and compared with the results for the bulk (experiment 2).

### FIB processing

For experiment 1, both edges of the 0.5-mm-diameter and 2.54-mm-long quartz template were polished to obtain good electrical and thermal contacts with the bismuth nanowire. Metal thin-film layers of Ti (100 nm) and Cu (1,000 nm) were then deposited on both polished end surfaces of the nanowire and template using an ion plating method. The resistance was measured using the two-wire method with an alternating current (AC) and a lock-in amplifier at precisely controlled (<1 mK) temperatures from 4.2 to 300 K achieved using a Gifford-McMahon (GM) cryocooler [34,35]. In the next step of the experiment, one side surface of the quartz template was removed by polishing until just before the bismuth nanowire was exposed, as shown in Figure 1b. The distance between the surface of the bismuth nanowire and the quartz template was less than 1 μm, as measured with a laser microscope. After removal of the quartz template, the sample was attached with adhesive onto a doped silicon (Si) wafer to prevent charge-up during FIB processing, with the polished surface upward. Ti (100 nm)/Cu (200 nm) thin-film layers were then deposited on the polished surface. The thin-film layers acted as electrodes and helped to prevent charge-up during FIB processing because the majority of the sample was quartz. This sample was installed into a dual-beam FIB-scanning electron microscope (SEM) apparatus (NB5000, Hitachi High-Technologies Ltd., Tokyo, Japan), and six electrical contacts were fabricated by FIB processing.

**Figure 2:**
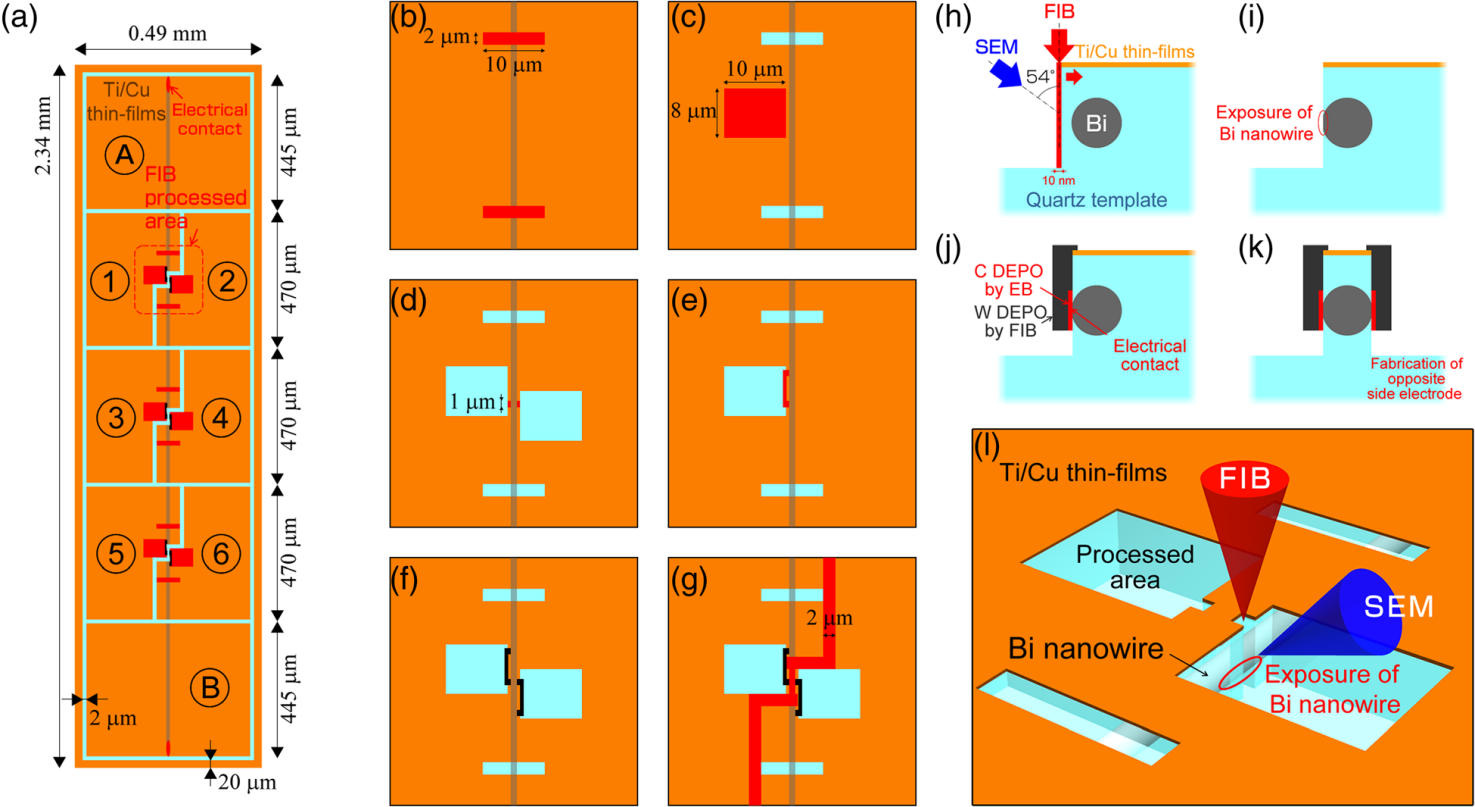
**Schematic diagrams for FIB processing to fabricate Hall measurement electrodes on a 521-nm-diameter bismuth nanowire. (a)** Overall view of the fabricated sample. **(b-g)** Procedure for the fabrication of electrodes by FIB. **(h-k)** Cross-sectional view during electrode fabrication. **(l)** 3-D view of processing with the dual-beam FIB-SEM.

Figure 2 shows schematic diagrams of the FIB processing used to prepare electrodes on the bismuth nanowires for the four-wire resistance and Hall measurements. The width and length of the quartz template were 0.49 and 2.34 mm, respectively. Eight parts of the electrodes are labeled with 1 to 6, and A and B in Figure 2a. Both ends of the bismuth nanowire at electrodes A and B are connected to Ti/Cu thin films after polishing because both ends of the nanowire are exposed by polishing. Electrical contacts at electrodes 1 to 6 were fabricated by FIB processing. We have previously established a technique to fabricate ohmic contact electrodes on the side surfaces of a bismuth nanowire for four-wire resistance measurement by ion beam sputtering and deposition of a thin film onto the surface of a nanowire in a quartz template using FIB [32]. An advanced technique was applied to fabricate electrodes for Hall measurement in this study. All FIB processing and fabrication was performed using a Ga ion beam accelerated at 30 kV. The bismuth nanowire was located at almost the center of the quartz template, so that the approximate position of the nanowire could be determined by coordinated positioning of the microscope with an accuracy of several micrometers. Firstly, two rectangular areas (2 × 10 μm^2^) on the quartz template were sputtered above the nanowire, using FIB as shown in Figure 2b, to determine the exact position of the bismuth nanowire with *ca*. 10-nm accuracy. Even if the quartz template covered the bismuth nanowire, the difference in the emission ratio of secondary electrons indicated where the bismuth nanowire was aligned [32,33]. Secondly, a rectangular volume of 8 × 10 μm^2^ and a depth of *ca*. 5 μm were removed at one side position of the nanowire, as shown in the Figure 2c. The side surface of the bismuth nanowire was then exposed with a width of 1 μm, and electrical contact to the bismuth nanowire was obtained using carbon film deposition by *in situ* reaction between the electron beam (EB) and phenanthrene (C_14_H_10_) gas, as shown in Figure 2d. The carbon electrode on the nanowire was connected to the Ti/Cu thin films deposited on the quartz template (Figure 2e) by a low electrical resistance tungsten (W) film that was deposited by reaction between the Ga ion beam and hexacarbonyltungsten (W(CO)_6_). Figure 2h,i,j,k shows schematic cross sections for the electrode fabrication process using FIB-SEM. The quartz template at the side area of the bismuth nanowire was already removed, as shown in Figure 2c. The remaining part of the quartz template was gradually removed with a very low current ion beam (10 nm wide) and at a very slow rate to carefully expose the bismuth nanowire and avoid damage to the nanowire. The surface was observed using SEM during removal of the quartz template; the SEM was located at tilt angle of 54° from the FIB. Figure 2l shows a 3-D schematic diagram of the process using dual-beam FIB-SEM. The Ga ion beam irradiation was stopped just after exposure of the bismuth nanowire, as shown in Figure 2i. Localized areas of the bismuth nanowire could be successfully exposed using this procedure. Carbon and tungsten electrodes were then deposited on the exposed surface of the bismuth nanowire, as shown in the Figure 2j. In a previous work, a Ga ion beam was used to deposit carbon electrodes on a bismuth nanowire; however, although the ion beam could deposit carbon film at a high deposition rate, it was confirmed that the heavy mass of Ga ions sputtered the components of the bismuth nanowire [32]. If all connections were produced by only carbon deposition, then electrical contact could not be obtained due to its high resistance. Therefore, a very thin carbon layer (*ca*. 100 nm thick) was deposited using the EB to minimize the resistance and prevent damage to the bismuth nanowire from the Ga ion beam irradiation during tungsten deposition. The thickness of the carbon deposition was determined by considering the resistance of carbon and the depth of Ga ion penetration (30 nm). It would be preferable that all electrical contacts be composed of only tungsten deposition; however, the FIB-SEM apparatus that was utilized in this experiment could not deposit tungsten using the EB. Therefore, a combination of carbon and tungsten was utilized for the electrodes on the bismuth nanowire. The opposite side electrode was also fabricated using the same procedure, as shown in Figure 2f,k. Almost all of the bismuth nanowire was not irradiated with the Ga ion beam because the bismuth nanowire was encapsulated within the quartz template. Finally, the electrodes were divided into two parts with a 2-μm-wide groove, as shown in Figure 2g, and all electrodes were divided into eight parts, as shown in Figure 2a.

**Figure 3:**
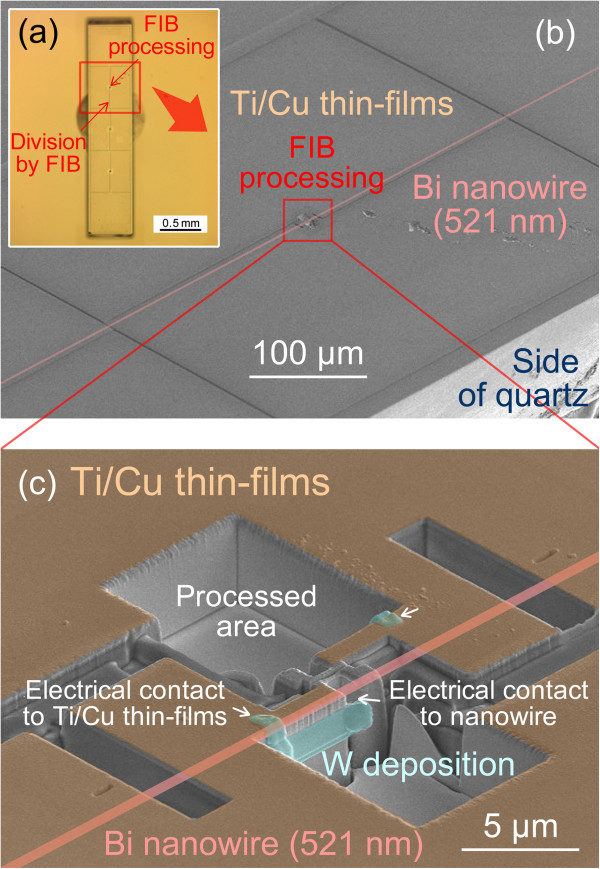
**Micrographs of electrodes fabricated on a 521-nm-diameter bismuth nanowire. (a)** Optical micrograph of the sample processed by FIB, **(b)** SEM micrograph of the electrical connections to the bismuth nanowire, and **(c)** magnified SEM micrograph of the FIB processed area.

**Figure 4:**
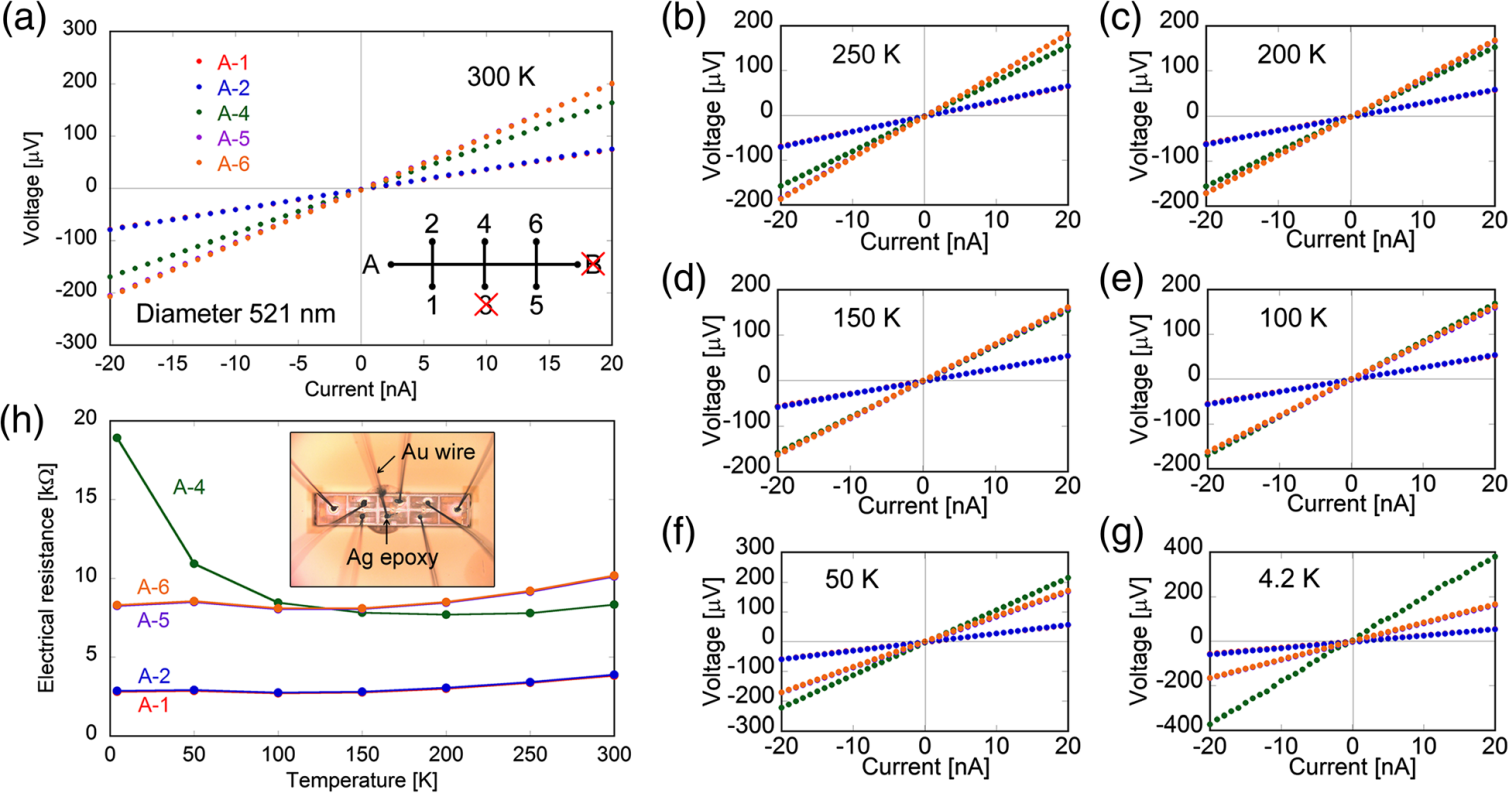
**Current–voltage characteristics for various electrode pairs on the 521-nm-diameter bismuth nanowire measured at various temperatures. (a)** 300, **(b)** 250, **(c)** 200, **(d)** 150, **(e)** 100, **(f)** 50, and **(g)** 4.2 K. **(h)** Temperature dependence of the electrical resistance evaluated from the *I*-*V* curves. The inset of (h) shows the fabricated sample used for the measurement.

Figure 3a shows an optical micrograph of the sample after FIB processing. The Ti/Cu thin films on the quartz template are divided into eight-part electrodes by FIB processing. Figure 3b,c shows SEM images of the electrical connections that formed between the bismuth nanowire and Ti/Cu thin films using FIB. The pink diagonal lines in Figure 3b,c indicate the approximate position of the bismuth nanowire embedded in the quartz template. Both side surfaces of the bismuth nanowire were connected to Ti/Cu thin films on the quartz template by tungsten deposition. The Ti/Cu thin films on the quartz template were divided by the groove formed using FIB to insulate each part. The connections of all electrodes were tested using a digital multimeter, and the electrodes were confirmed to be successfully fabricated on the bismuth nanowire by FIB processing. The nanowire sample mounted on a Si wafer was fixed to an alumina plate (23 × 16 × 0.5 mm^3^) with an adhesive, and gold (Au) lead wires were attached to all electrodes using silver (Ag) epoxy, as shown in the inset of Figure 4h. Au wires were connected to the measurement system through electrodes on the alumina plate. The contacts of the electrodes on the nanowire were evaluated by measuring the relationship between the current passed and the voltage.

## Results and discussion

### Current–voltage characteristics

Figure 4a,b,c,d,e,f,g shows current–voltage (*I*-*V*) characteristics for various combinations of electrodes on the bismuth nanowire measured at 300, 250, 200, 150, 100, 50, and 4.2 K. The measurement was performed with a direct current (DC) from −20 to +20 nA. The electrodes labeled as B and 3 were broken during a decrease in the temperature. The *I*-*V* characteristics of all the electrodes are clearly linear over the entire temperature range examined, which indicates that the electrodes fabricated by FIB were ohmic contacts. The resistance values agreed well for pair combinations of A-1 and A-2, A-5 and A-6 because the distances between the electrodes were the same. Figure 4h shows the temperature dependence of the electrical resistance evaluated from these *I*-*V* characteristics. The resistance increased in the order of A-1, A-2 < A-4 < A-5, A-6 at 300 K depending on the distance between electrodes. However, the resistance of A-4 became larger than that of A-5 and A-6 at less than 100 K. The increase in the resistance of A-4 with decreasing temperature may be due to the long length of the carbon electrode on the nanowire, although it did not significantly influence the four-wire method.

### Resistivity measurement of 521-nm-diameter nanowire

**Figure 5:**
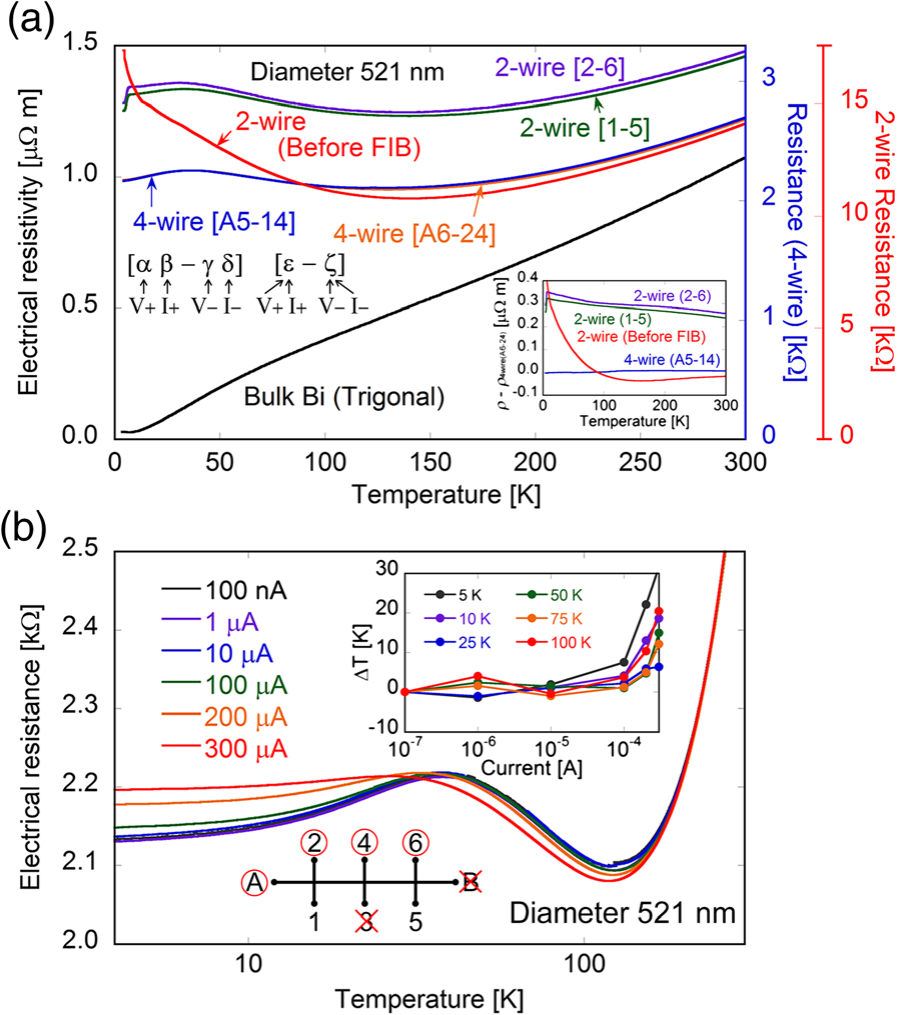
**Temperature dependence of the resistivity of the 521-nm-diameter bismuth nanowire. (a)** Temperature dependence of the resistivity for the bismuth nanowire measured with various electrode combinations. The inset of (a) shows the temperature dependence of the difference in resistivity from the four-wire method [A(*I*_+_)6(*I*_−_)-2(*V*_+_)4(*V*_−_)]. **(b)** Temperature dependence of the resistivity for the bismuth nanowire measured at various electric currents. The inset of (b) shows the dependence of the temperature on the current from that at 100 nA. The numbers and letters which denote electrodes utilized for resistance measurements are shown with respect to the following rules: [α(*I*_+_)β(*I*_−_)-γ(*V*_+_)δ(*V*_−_)] for the four-wire method and [ϵ(*I*_+_,*V*_+_)-ζ(*I*_−_,*V*_−_)] for the two-wire method.

The temperature dependence of the resistivity was measured from 4.2 to 300 K at 10 nA, and the two-wire and four-wire resistance measurements were compared. Figure 5a shows the temperature dependence of the electrical resistivity for the bismuth nanowire measured by the AC method with various pairs of electrodes. The resistance measured by the two-wire method before FIB processing, by the two-wire method with various pairs of electrodes fabricated by FIB, by the four-wire method with fabricated electrodes, and that for bulk bismuth are also shown in the figure. The temperature dependence of the bismuth nanowire was different from that of bulk bismuth, especially in the low temperature range, which was caused by the limitation on the carrier mean free path, as reported previously [15,22]. The results showed that the resistivity from the two-wire method before FIB processing was close to that from the four-wire method at 300 K; however, the difference became more apparent with decreasing temperature. The four-wire method is generally the most accurate to measure resistivity; therefore, differences from the four-wire resistivity using electrodes A(*I*_+_)6(*I*_−_)-2(*V*_+_)4(*V*_−_) are shown in the inset of Figure 5a. The resistivity by the two-wire method before FIB processing increased with decreasing temperature, which indicates that the contact resistance is not negligible, even if the resistance of the nanowire was extremely large, such as over the kilo-ohm level. Although many researchers have reported the resistivity of bismuth nanowires measured by the two-wire method, due to difficulty of the four-wire method with a very small diameter nanowire [6-12], the accuracy of the resistivities measured by the two-wire method should be carefully considered. The resistivities determined by the two-wire method using 1(*I*_+_,*V*_+_)-5(*I*_−_,*V*_−_) and 2(*I*_+_,*V*_+_)-6(*I*_−_,*V*_−_) electrodes became larger than those determined by the four-wire method, which implies that the contact resistance of the electrodes fabricated by FIB is not negligible. The temperature dependence of resistivity showed a sharp drop at very low temperature (*ca*. 3.7 K), which was caused by the superconductivity transition of the tungsten deposit fabricated by FIB. Although the superconductivity transition temperature of pure tungsten is 0.01 K, it was already reported that the transition temperature of amorphous tungsten including carbon became larger than that of pure tungsten [36]. Therefore, if the electrodes are fabricated with only the tungsten deposition, ideal superconductivity electrodes could be applied for measurement at very low temperature. Figure 5b shows the temperature dependence of the resistivity for the bismuth nanowire measured at various electric currents from 100 nA to 300 μA using the four-wire method with the A(*I*_+_)6(*I*_−_)-2(*V*_+_)4(*V*_−_) electrodes. The inset of Figure 5b shows the dependence of the temperature variation on the current from the temperature at 100 nA (Δ*T*) due to joule heating calculated from the temperature coefficient and the difference in the resistance. It was confirmed that obvious temperature variation was shown to be higher than 100 μA. Thus, electric current up to 10 μA can be applied to the 521-nm-diameter bismuth nanowire for Hall measurements. It is surprising that such a high current density of 47 A/mm^2^ could be applied to the very narrow diameter nanoscale wire. This result indicates that almost all of the joule heat from the nanowire is absorbed into the surrounding quartz template, which possesses much larger heat capacity than the bismuth nanowire, as reported in [37]. This is an advantage of covering the nanowire with the template because the high current makes it easier to measure the Hall voltage of the bismuth nanowire.

### Hall measurement of 4-μm-diameter microwire

**Figure 6:**
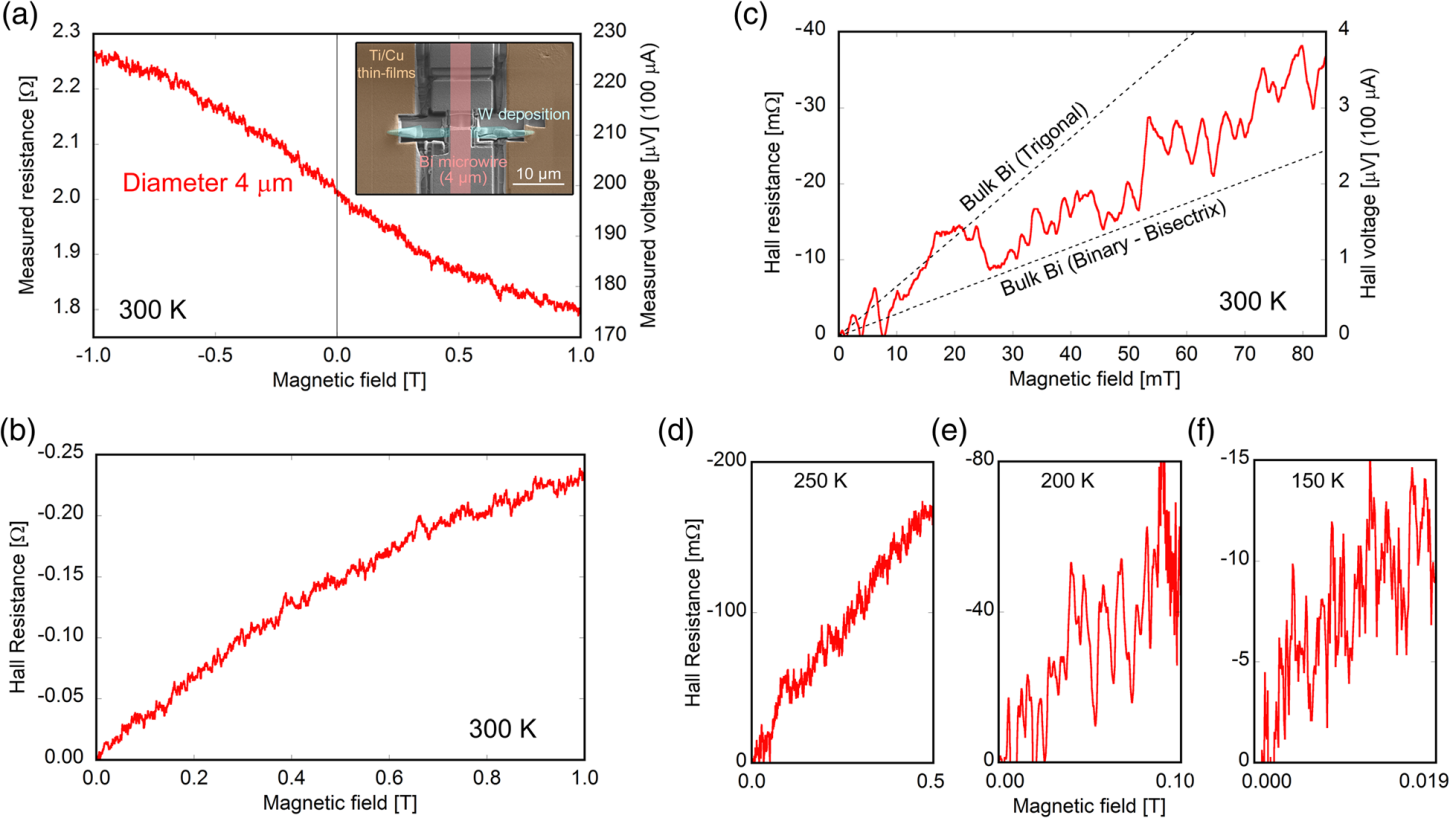
**Magnetic field dependence. (a)** Magnetic field dependence of the resistance for the 4-μm-diameter bismuth microwire with the current at 100 μA. The inset of (a) shows a SEM micrograph of the electrodes fabricated by FIB on the bismuth microwire. Magnetic field dependence of the Hall resistance evaluated from the measured resistance **(b)** in the range from 0 to 1 T and **(c)** in the low magnetic field range from 0 to 85 mT with the expected values for bulk bismuth in two directions. **(d-f)** Magnetic field dependence of the Hall resistance at 250, 200, and 150 K.

Hall measurements were conducted for the bismuth microwire sample within the quartz template (experiment 2) to determine whether Hall measurements could be successfully performed and compared with the results for bulk bismuth. A 4-μm-diameter and 3.68-mm-long bismuth microwire sample was fabricated for Hall measurements, as shown in Figure 1c. Electrodes on the bismuth microwire were fabricated in the same way as that for experiment 1. The inset of Figure 6a shows a SEM micrograph of the electrodes fabricated on the bismuth microwire. The vertical red line in the center indicates the position of the bismuth microwire. The two points on the surface of the microwire were connected to Ti/Cu thin films with tungsten deposition. Hall measurements were performed under application of negative and positive magnetic fields generated with a superconducting magnet. The Hall resistance was measured by the AC method in the frequency range from 0.2345 to 11.234 Hz and was dependent on the temperature because the contact resistance of electrodes changed with the temperature. The contact resistance increases with decreasing temperature; therefore, lower frequency was required to reduce the phase lag. Figure 6a shows the magnetic field dependence of the measured resistance from −1 to 1 T at 300 K. The measured resistance was the sum of the Hall resistance and diagonal resistance, and the diagonal resistance could not be ignored due to the low carrier density of semi-metallic bismuth. The Hall resistance could be extracted from the measured resistance because the Hall resistance is an odd function and the diagonal resistance is an even function for the magnetic field. Figure 6b shows the Hall resistance evaluated from the measured resistance in the range from 0 to 1 T, and Figure 6c shows the result in the low magnetic field range from 0 to 85 mT, considering a linear relationship between the Hall resistance and magnetic field [38,39]. The dashed lines indicate the values for bulk bismuth, where the upper is for the trigonal direction and the lower is for the binary-bisectrix plane. The measured Hall resistance is in the same range as that for bulk bismuth, which confirms that the Hall measurements of the bismuth microwire were successful. Figure 6d,e,f shows the magnetic field dependence of the Hall resistance at 250, 200, and 150 K.

**Figure 7:**
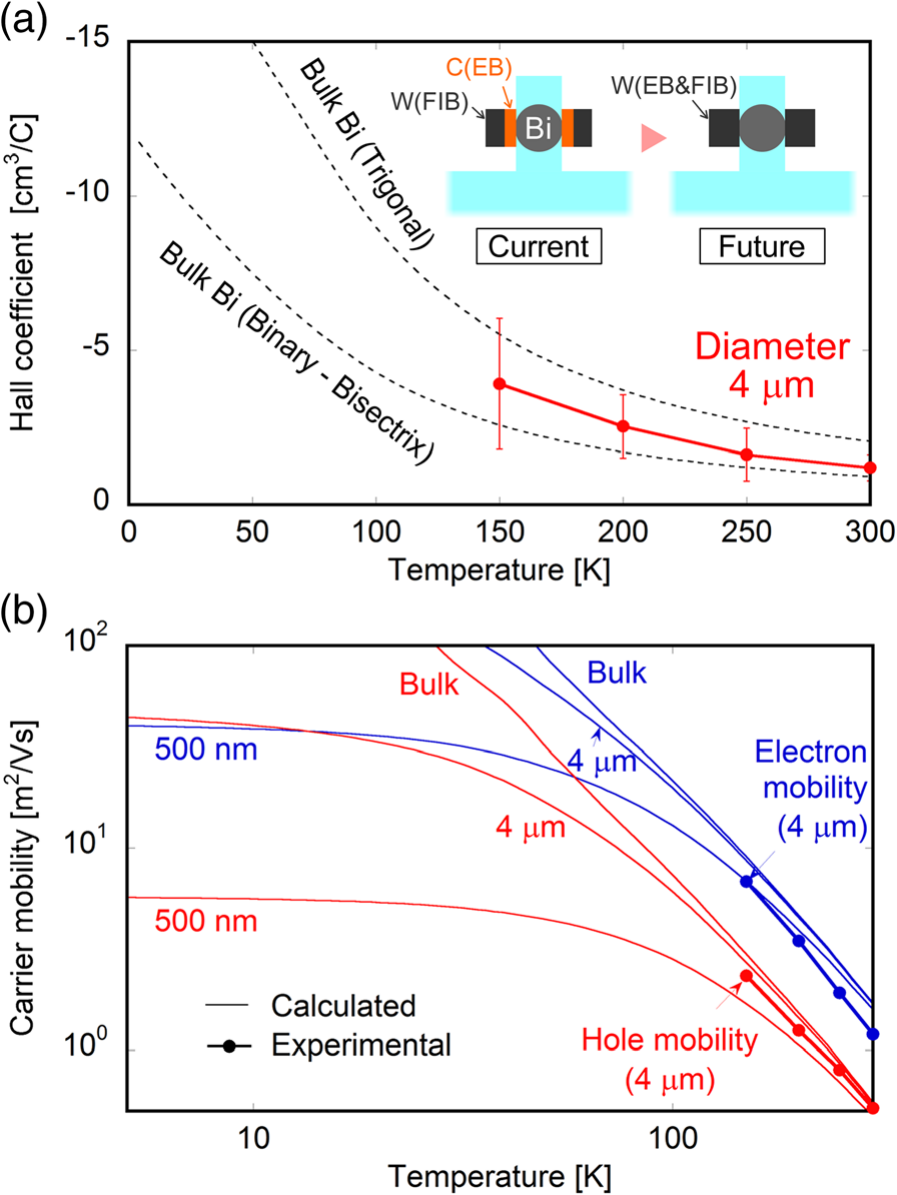
**Temperature dependence of Hall coefficient and carrier mobility. (a)** Temperature dependence of the measured Hall coefficient for the 4-μm-diameter bismuth microwire and the expected values for bulk bismuth in two directions. **(b)** Temperature dependence of carrier mobility evaluated from the Hall coefficient and the expected values of bulk bismuth for the binary-bisectrix direction.

Figure 7a shows the temperature dependence of the Hall coefficient for the 4-μm-diameter bismuth microwire calculated from the magnetic field dependence of the Hall resistance using a least-squares method and that for bulk bismuth in two directions. The Hall coefficient (*R*_H_) was calculated from $R_{H}=R_{\text{Hall}}\frac{\mathrm{d\pi}}{4B}$[33], where *R*_Hall_, *d*, and *B* are the Hall resistance, the wire diameter, and the magnitude of the magnetic field, respectively. The measurement was successfully performed from 150 to 300 K, and the result was in the same range as that for bulk bismuth. However, Hall measurement became difficult in the low temperature range due to a very low signal-to-noise (S/N) ratio of the Hall voltage caused by the high contact resistance of the carbon electrodes fabricated by FIB. This result implies that carbon electrodes are not appropriate for this measurement due to their high resistance. Therefore, we are planning to fabricate electrodes that consist only of tungsten, as shown in the inset of Figure 7a; this will be achieved using another FIB apparatus that is equipped with an EB for tungsten deposition. Figure 7b shows the temperature dependence of the electron (μ_e_) and hole (μ_h_) mobilities estimated from the Hall coefficient and the electrical resistivity according to the following equations that apply the charge-neutrality condition [38]:

(1) $$R_{H}=\frac{r_{H}}{\mathrm{en}}\frac{\mu_{h}^{2}-\mu_{e}^{2}}{{\left(\mu_{h}+\mu_{e}\right)}^{2}}$$

and

(2) $$\frac{1}{\rho }=\mathrm{en}\;\left(\mu_{h}+\mu_{e}\right)$$

where *r*_H_, *e*, *ρ*, and *n* are the Hall factor, the elementary charge, the electrical resistivity, and the carrier density, respectively. The resistivity measured for another 4-*μ*m-diameter microwire was utilized for *ρ*, and the carrier density of bulk bismuth from [2] was utilized in Equation 2. The value of *r*_H_ was 1.18, because the scattering process of bismuth is assumed to be acoustic phonon scattering [38]. Literature values of the carrier mobilities for bulk bismuth [40,41] and those expected for the 4-μm microwire and 500-nm nanowire calculated using the mean free path limitation model [23] and assuming the bisectrix direction are also represented in Figure 7. Unfortunately, the crystal orientation of the bismuth microwire was not measured because the sample was fabricated as a trial. It could be confirmed that both the experimental and calculated results for the 4-μm-diameter bismuth microwire and those for bulk bismuth were in the same range at over 150 K, which indicates that the carrier mobilities of the bismuth microwires were successfully evaluated by the Hall measurement. In addition, the carrier mobility of the 500-nm nanowire was clearly different from that of bulk bismuth and the 4-μm microwire over the entire temperature range. Therefore, we are planning to measure the carrier mobilities of bismuth nanowires with diameters of several hundred nanometers after solving the problem of the high contact resistance electrodes fabricated by FIB. This problem could possibly be solved by using electrodes that consist only of tungsten, rather than a combination of high-resistance carbon and tungsten. Thus, a decrease of the carrier mobility in bismuth nanowires and the dependence on the diameter should be revealed by Hall measurements in a future work.

## Conclusions

We have successfully fabricated ohmic contact electrodes for measurement of the four-wire resistance and Hall voltage in an individual single-crystal bismuth nanowire with a diameter of 521 nm and a length of 2.34 mm covered with a 0.5-mm-diameter quartz template. FIB processing was utilized to expose the side surfaces of the bismuth nanowire, and carbon and tungsten electrodes were deposited on the bismuth nanowire *in situ* to obtain electrical contact without severe damage to the bismuth nanowire. Oxidation of the bismuth nanowire could be prevented because the bismuth nanowire was covered with the quartz template and all the electrode fabrication procedures were performed under high vacuum. The measured *I*-*V* characteristics confirmed that ohmic contacts were obtained over the entire temperature range from 4.2 to 300 K. This result indicates that the electrodes on the bismuth nanowire could be successfully fabricated by FIB processing with suitable contacts for four-wire resistance and Hall measurements. Furthermore, measurement of the temperature dependence of the four-wire resistance was successfully performed for the bismuth nanowire using the fabricated electrodes from 4.2 to 300 K. A difference between the results for the two-wire and four-wire resistances was observed, which indicates that the contact resistance was not negligible, even if the resistance of the nanowire was extremely large and over several kilo-ohms. Although there have been many reports on the resistivity measured using the two-wire method, we must carefully consider whether resistivities measured by the two-wire method are correct. Furthermore, Hall measurements were also conducted on a 4-μm-diameter bismuth microwire, and the evaluated carrier mobility was in good agreement with that for bulk bismuth, which indicates that the carrier mobility of the bismuth microwire in the quartz template could be successfully measured with this technique. Hall measurements were difficult in the low temperature range due to the high contact resistance of the carbon electrodes employed. Therefore, we are planning to fabricate electrodes that consist of only tungsten and to measure the carrier mobilities of bismuth nanowires with diameters of several hundred nanometers.

## Competing interests

The authors declare that they have no competing interests.

## Authors’ contributions

MM conducted the experiments, polishing, FIB processing, and resistance and Hall measurements and drafted the manuscript. YH guided the idea and the experiments and revised the manuscript. All authors have read and approved the final manuscript.

## Authors’ information

MM is a Ph.D. candidate under Associate Professor YH in the Department of Engineering, Saitama University, Japan.
